# Posterior Pericardiotomy: Historical Perspective, Current Techniques, and Clinical Outcomes

**DOI:** 10.1016/j.atssr.2026.03.006

**Published:** 2026-03-26

**Authors:** Edward Hauptmann, Erin Hoover, Raghav Chandra, Madhuri Nagaraj, Paige Deblieux, Jasmina Ehab, Suresh Keshavamurthy

**Affiliations:** 1 Department of General Surgery, University of Texas Southwestern Medical Center, Dallas, Texas; 2 Department of Cardiothoracic Surgery, University of California San Francisco, San Francisco, California; 3 Department of General Surgery, University of Colorado, Aurora, Colorado; 4 Department of Cardiothoracic Surgery, University of Texas Southwestern Medical Center, Dallas, Texas

## Abstract

**Background:**

Cardiac tamponade and postoperative atrial fibrillation remain significant challenges after cardiac surgery, contributing to higher morbidity, mortality, and health care costs. Posterior pericardiotomy (PP) has been used to reduce these complications. This review summarizes the history, technique, and clinical evidence for PP.

**Methods:**

We identified recent studies related to PP to perform a narrative overview of its indications, techniques, and clinical outcomes. This was supplemented with historical context from cardiac surgeons who were early adopters of the procedure.

**Results:**

Evidence demonstrates that PP reduces postoperative pericardial effusion, tamponade, and postoperative atrial fibrillation by improving pericardial drainage, preventing tamponade, and secondarily decreasing pericardial inflammation and oxidative stress.

**Conclusions:**

Despite receiving a class 2a recommendation in recent American College of Cardiology guidelines for atrial fibrillation prevention, PP is not widely performed. Given its simplicity, safety, and effectiveness, wider use of PP could improve outcomes after cardiac surgery.

**In Short**

▪ Posterior pericardiotomy reduces postoperative cardiac tamponade and atrial fibrillation.
▪ Posterior pericardiotomy received a class 2a recommendation for atrial fibrillation prevention but is not widely performed.

Posterior pericardiotomy (PP) was introduced as a means of reducing the incidence of postoperative pericardial effusion and cardiac tamponade. Cardiac tamponade occurs in up to 5.2% of cardiac surgeries and contributes to prolonged hospitalization and surgical reintervention.^1^^,^^2^ PP allows drainage of blood from the pericardial space to the left pleural space, decreasing the chance for cardiac tamponade.^3^ Retained pericardial blood is a source of inflammation and oxidative stress on the heart and has been implicated in the development of postoperative atrial fibrillation (POAF), which affects as many as 33% of patients.^4^ Importantly, POAF has been found to increase rates of mortality, stroke, acute kidney injury, hospital length of stay, hospital costs, and hospital readmission.^5^

Randomized trials and meta-analyses support the use of PP to mitigate the risk of pericardial effusion, tamponade, and POAF.^6^^,^^7^ Whereas PP has garnered renewed academic interest, adoption into practice remains limited. Accordingly, this review aimed to clarify the physiologic rationale for the procedure, to describe its origins and techniques, and to discuss applications and clinical outcomes to contextualize its current role in cardiac surgery and to identify priorities for future investigation.

## Material and Methods

A literature review was performed using the electronic databases PubMed and MEDLINE. Search terms included *posterior pericardiotomy*, *cardiac tamponade*, and *postoperative atrial fibrillation*. This resulted in meta-analyses, randomized controlled trials, guideline statements, and observational studies describing the indications, outcomes, and variations of the procedure. To supplement our narrative review’s historical perspective, we consulted cardiac surgeons from multiple institutions worldwide, primarily former colleagues who were known early adopters of PP, to gain insight into the initial indications and adoption of PP.

### Surgical Techniques

**Figure 1:**
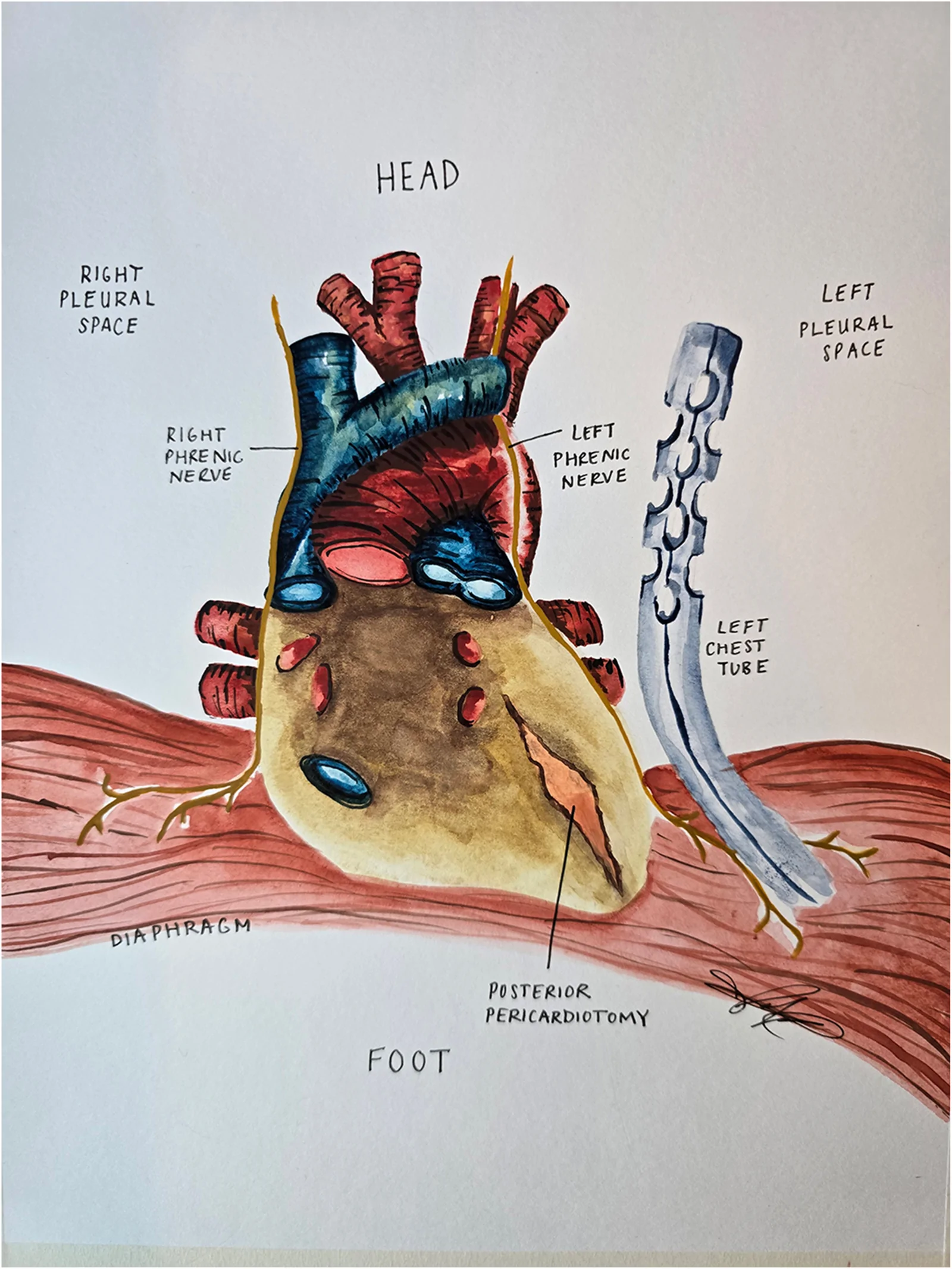
Traditional posterior pericardiotomy location.

**Figure 2:**
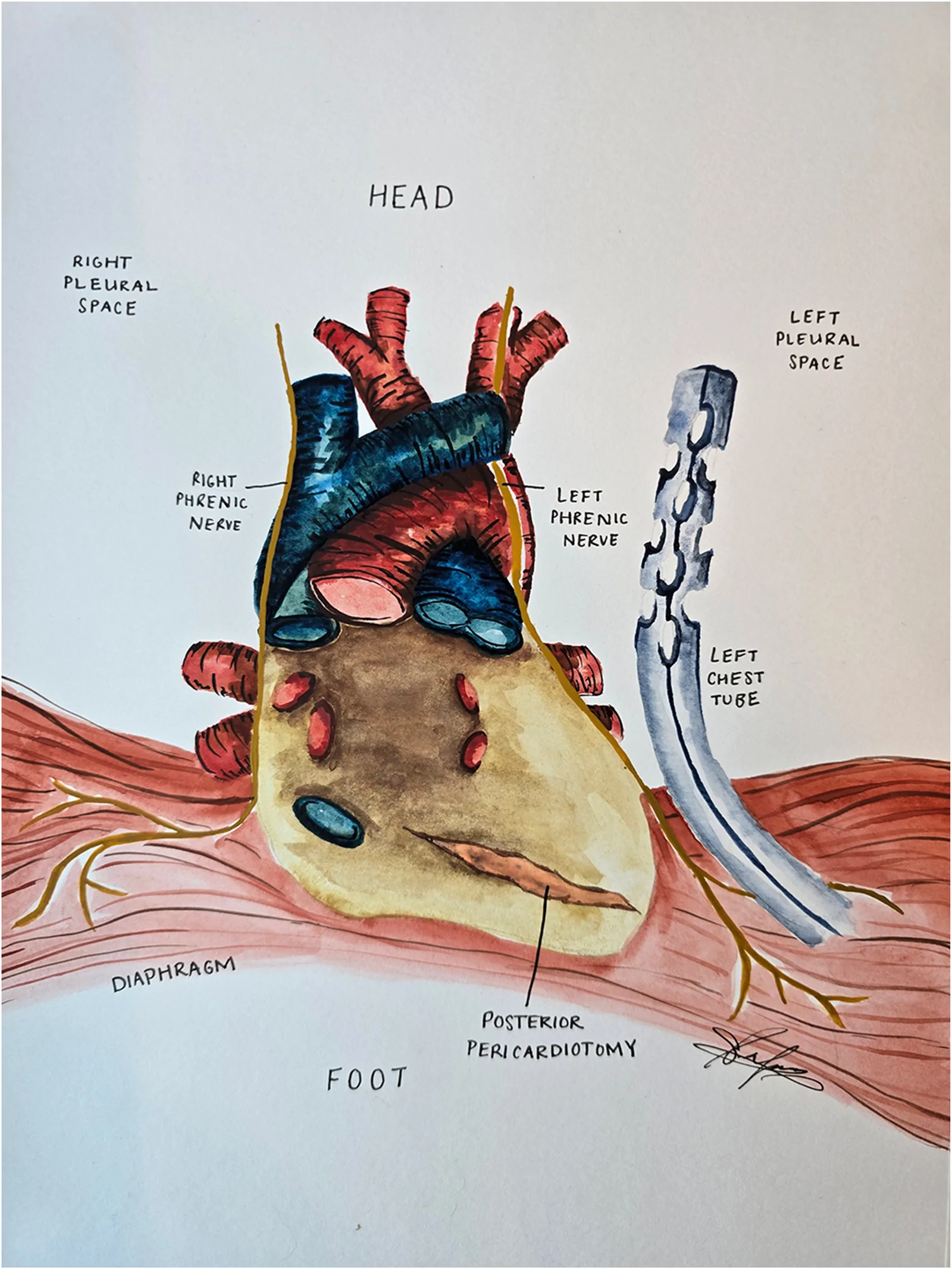
Posterior pericardiotomy with a transverse orientation.

The PP technique was first reported in 1995 by Mulay and coworkers^3^ and later described in further detail by Gaudino and coworkers^6^ in 2021. The heart is lifted out of the pericardial well to expose the posterior pericardium. A 4- to 5-cm incision is created posterior to the phrenic nerve between the left inferior pulmonary vein and the diaphragm, and a left pleural chest tube is placed. This is traditionally a longitudinal incision but may be performed in a more transverse orientation (Figures 1, 2). The pleural tube does not need to be placed through the pericardiotomy, and at this time, the optimal tube type remains unclear.

**Figure 3:**
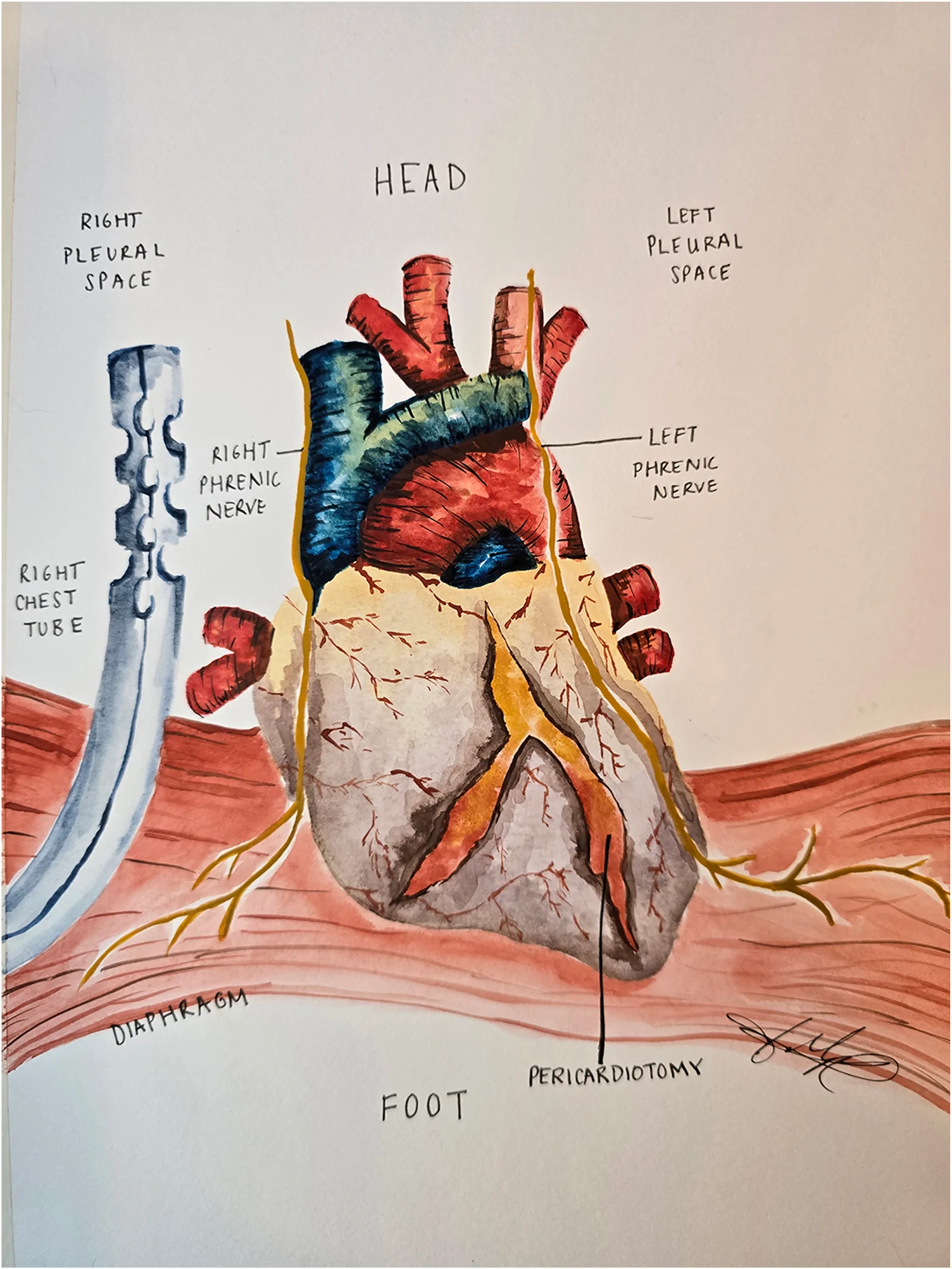
Right-sided extension of anterior pericardiotomy with pleural tube drainage.

**Figure 4:**
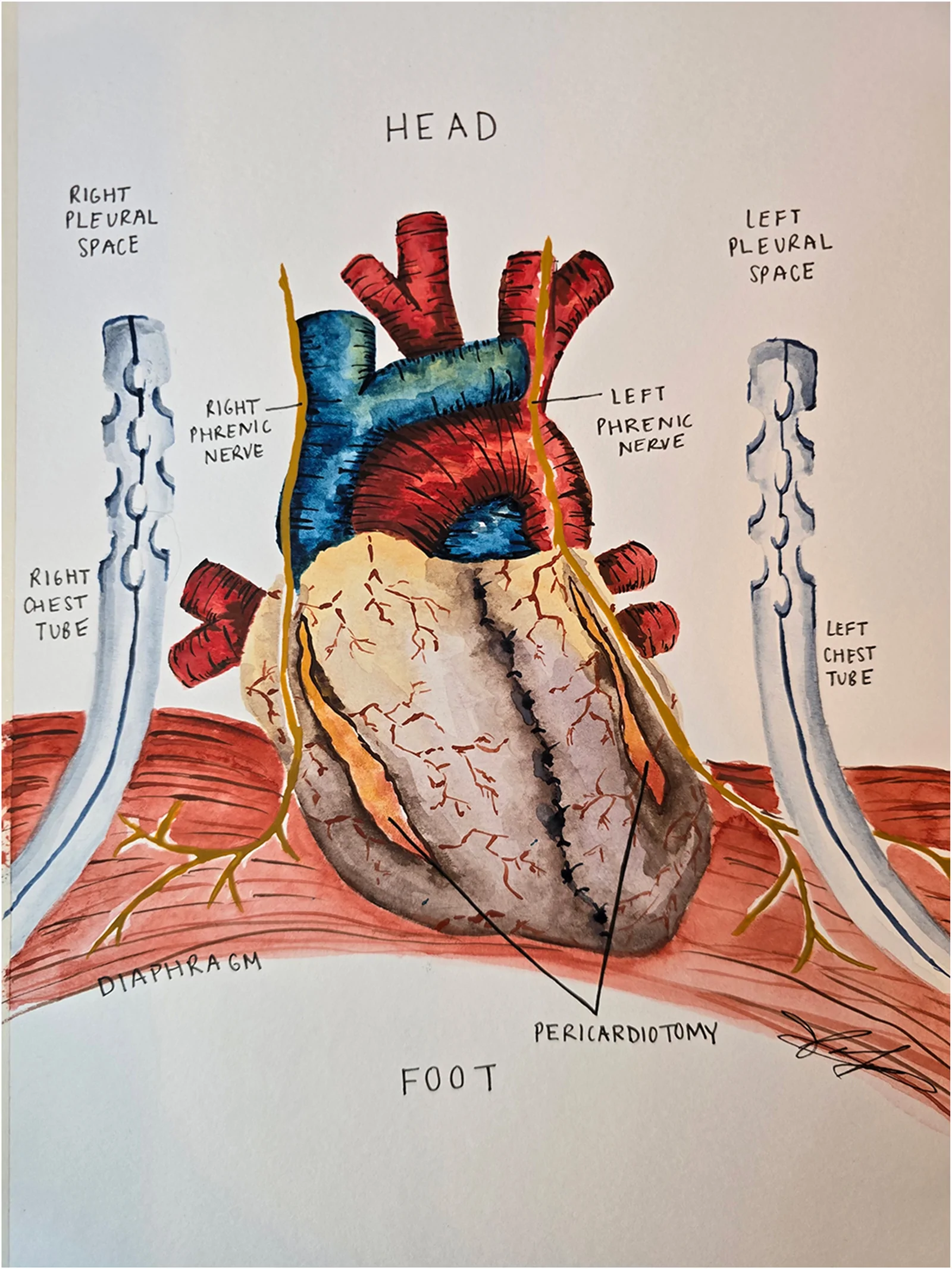
Bilateral posterior pericardiotomies with anterior pericardial closure.^9^

Alternative approaches include extending the anterior pericardiotomy to the right, medial to the phrenic nerve (Figure 3) or a right pericardial window, created by incising the pericardium 4 cm longitudinally and 1 cm wide, 2 cm above the phrenic nerve adjacent to the right thorax.^8^ To reduce anterior adhesions, which is particularly beneficial if the patients should require a redo sternotomy, some surgeons report that closing the anterior pericardium and creating bilateral posterior longitudinal pericardiotomies facilitate drainage and relieve anterior pericardial tension while providing a protective tissue layer over the heart (Figure 4).^9^ Regardless of technique, any posterior pericardial incision should be documented as this may cause adhesions between the pericardium or the posterior surface of the heart and surrounding thoracic structures, potentially affecting future cardiothoracic operations, including minimally invasive approaches.

Currently, robust data exist only for the left PP technique. Thus, it is not certain how efficacious alternative approaches are or how they compare with left PP. These techniques have been discussed to illustrate the range of pericardial drainage methods that have been employed and to highlight the need for future studies to evaluate their effectiveness.

## Results

### History of Posterior Pericardiotomy

PP has been used in practice internationally since the 1980s. We spoke with Dr Sudhanshu Bhattacharya, a senior cardiac surgeon based in Mumbai, India. Dr Bhattacharya trained in Milwaukee, Wisconsin, in 1980-1981 under the renowned cardiac surgeon Dr Dudley Johnson, who routinely employed PP after cardiac surgery to prevent cardiac tamponade. In addition, Dr Johnson believed that this procedure allowed earlier removal of mediastinal drains, decreasing the risk of mediastinitis. Dr Bhattacharya carried his experience with PP to Mumbai, where he incorporated the technique into his practice at KEM Hospital.

**Figure 5:**
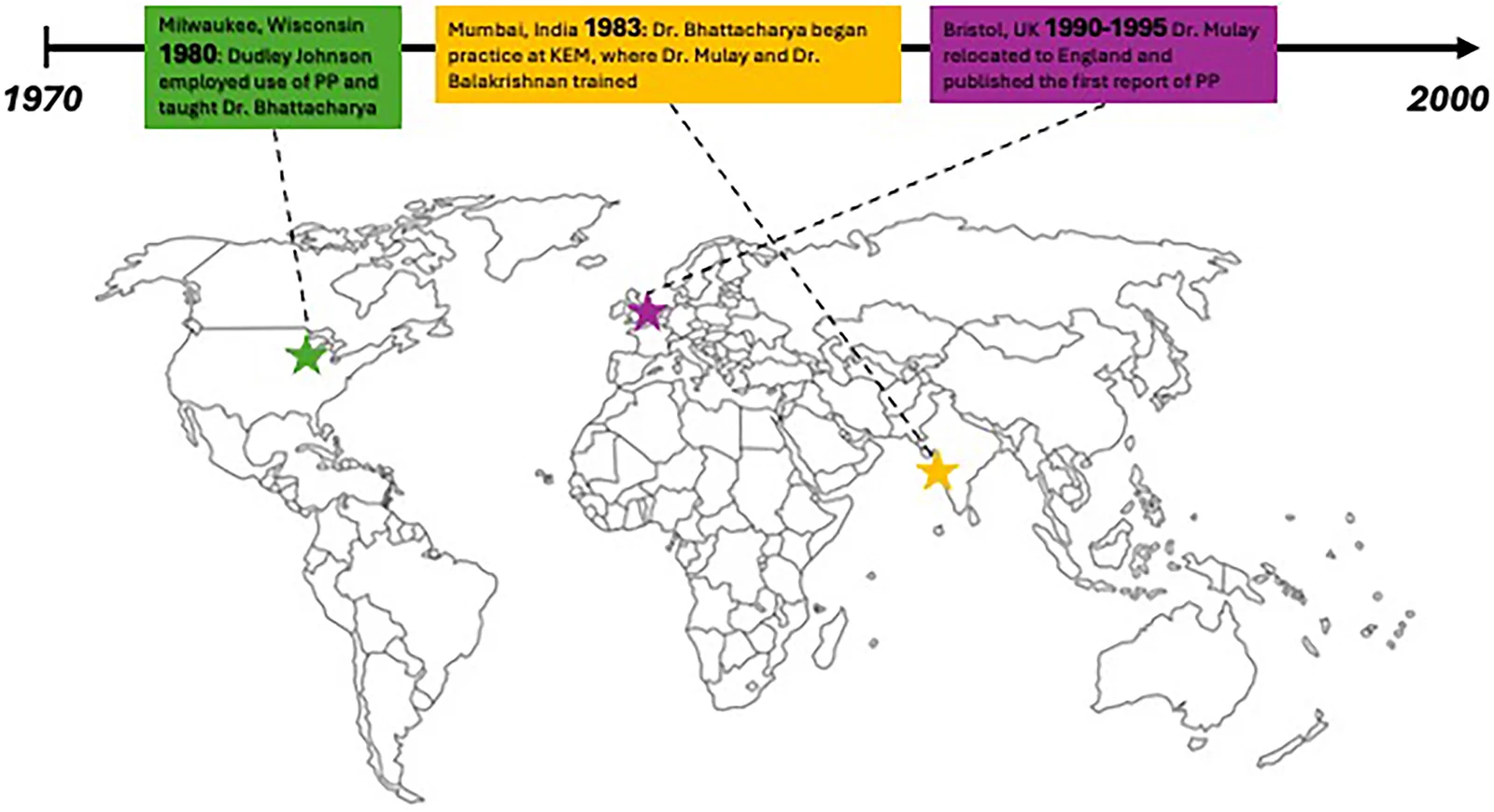
History of posterior pericardiotomy (PP) timeline.

Dr KR Balakrishnan and Dr Anvay Mulay also trained at KEM Hospital and could have been exposed to the technique through collaboration with Dr Bhattacharya. Dr Balakrishnan continued using PP in Chennai, India, where Dr Keshavamurthy was introduced to it in 1994. However, at this point, no outcome data were formally recorded, and all observations were purely anecdotal. Dr Mulay relocated to Bristol, United Kingdom, where he became the first surgeon to formally publish clinical outcomes associated with PP in 1995, demonstrating a reduction in pericardial effusion and supraventricular arrhythmias (Figure 5).^3^ The early adopters of PP who remain in practice are still using this technique.

Victor and Kabeer^10^ reported a case in which a patient with congenital absence of the pericardium underwent uncomplicated repair of an atrial septal defect with placement of a single chest tube in the left pleural space. The authors modified their technique to decrease the amount of chest tubes required. Instead of routinely placing mediastinal drains, they created a large right pericardial window and opened the pleura, creating a continuous pericardial-pleural-mediastinal space drained by a single pleural tube. This approach reportedly simplified patient transport and postoperative management by decreasing the need for mediastinal drains, which were placed only if excessive drainage was anticipated and require earlier removal than pleural tubes, and by potentially shifting infectious complications from mediastinitis to empyema, which is more readily managed with standard chest drainage.^10^ Notably, this report was purely observational and did not include quantitative outcome data.

In the 2000s, multiple case reports and prospective studies followed with similar results to the data reported by Mulay and coworkers. Growing international use during this period set the stage for larger randomized trials and meta-analyses evaluating the impact of PP on clinical outcomes, including the PALACS trial, which is the largest clinical trial to date studying the effects of PP.^6^^,^^11^

### Pathophysiologic Basis of Posterior Pericardiotomy

Observational and prospective studies have confirmed an association between postoperative pericardial effusions and the development of POAF.^4^ Oxidative breakdown products of retained blood in the pericardial space, operative trauma, and ischemia-reperfusion injury from cardiopulmonary bypass lead to a proinflammatory state.^12^ Hemolysis of intrapericardial blood produces reactive oxygen species. Local recruitment and activation of neutrophils and monocytes amplify the oxidative stress affecting the atrial surface, triggering POAF.^13^ Thus, the indication for PP is to provide a drainage pathway to shunt fluid from the pericardial space to the pleural space to reduce the incidence of postoperative pericardial effusions and tamponade, secondarily decreasing intrapericardial inflammation, decreasing the incidence of POAF.^3^

In addition to retained blood products in the pericardium, other variables have been linked to POAF. A large retrospective analysis by Rathnayake and coworkers^14^ from 2008 to 2022 identified that increased age, obesity, heart failure, and need for reoperation increase the risk of POAF. Hypertension, myocardial infarction, hypercholesterolemia, tobacco use, valvular heart disease, enlarged left atrium, left ventricle dysfunction, and structural heart disease also predispose to development of POAF.^15^ Recognizing these preoperative factors associated with POAF may help identify patients who may have increased benefit from PP.

### Other Applications of Posterior Pericardiotomy

Another population in which to consider PP is the pediatric population. A retrospective study by Simsek and coworkers^16^ reviewed 229 pediatric cardiac surgeries that included PP performed between 2021 and 2023. They found that prophylactic PP effectively prevented early and late cardiac tamponade in all cases, and anterior mediastinal drains were removed in 1.9 ± 0.4 days; however, this study did not include a control group. PP has also been used to allow chest closure in situations such as extracardiac Fontan procedures and size mismatch in cardiac transplantation, particularly in children with single-ventricle disease.^17^ This is especially useful in developing countries where pediatric donor organs are scarce. In addition, in patients with reduced pericardial space, PP may be used to expand the pericardial cavity, facilitating donor heart accommodation and chest closure.^17^ Of note, the population eligible for PP has limitations as most studies excluded patients with a prior thoracic or cardiac surgery, dense pleural adhesions, or left ventricular aneurysm, reducing generalizability.^18^

### Clinical Outcomes and Comparative Studies

The 1995 study by Mulay and coworkers,^3^ a single-center prospective study of 100 patients undergoing coronary artery bypass grafting (CABG), compared outcomes between those who received PP and those who did not. The PP group experienced significant decreases in pericardial effusion (8% vs 40%; *P* < .0005) and supraventricular arrhythmias (8% vs 36%; *P* < .005), with no evidence of phrenic nerve palsy or clinically significant increases in left pleural effusion.^3^

Since then, several meta-analyses have reported the efficacy of PP in decreasing POAF and pericardial effusion in CABG patients.^18^, ^19^, ^20^ The PALACS trial included 420 patients undergoing coronary, aortic valve, or aortic surgery and found that PP was associated with significantly lower risk of atrial fibrillation vs controls (17% vs 32%) and lower incidence of pericardial effusion (12% vs 21%).^6^ Following this, a meta-analysis from 18 randomized controlled trials reported that PP as part of CABG or valve surgery was associated with significantly lower risk of cardiac tamponade, early pericardial effusion, and POAF, with no difference in postoperative mortality, reoperation for bleeding, or need for postoperative balloon pump support.^20^ A 2023 meta-analysis found lower POAF incidence with PP compared with controls (11.7% vs 23.7%; odds ratio [OR], 0.49; 95% CI, 0.38-0.61; *P* < .001) as well as reduced pericardial tamponade (OR, 0.18; 95% CI, 0.10-0.33).^21^ PP has also been shown to specifically decrease late cardiac tamponade in patients who undergo valve replacement surgery, a population that is at relatively higher risk for this complication. One study reported that late tamponade occurred in 0% of patients who underwent PP and 5.2% of patients who did not (*P* = .015).^1^ This protective benefit is also seen in CABG patients. In the retrospective cohort analysis performed by Rathnayake and coworkers,^14^ of the 807 patients who received PP, none had tamponade, whereas 16 of 1333 in the control group had tamponade (0% vs 1.2%; *P* < .05), with no increase in postoperative bleeding. In addition, Fawzy and coworkers^22^ performed a multicenter, randomized controlled trial that displayed similar improvements in POAF (13% vs 30%; *P* = .003) and pericardial effusion (15% vs 53%; *P* < .001) and a shorter time to return to sinus rhythm (1.54 vs 3.66 days; *P* = .01) in patients in whom POAF developed who underwent PP. Pleural tube output was higher in the PP group, but this did not affect hospital length of stay.^22^ As previously mentioned, some surgeons believe that PP allows earlier removal of mediastinal tubes, potentially decreasing the incidence of mediastinitis. However, a 2025 meta-analysis found that postoperative day 1 removal increased pericardial effusion (relative risk, 1.85; *P* = .0105) without affecting intensive care unit stay, mortality, POAF, or infection, suggesting that PP may not affect timing of tube removal but can mitigate pericardial effusion risk.^23^

Interestingly, a 2015 randomized controlled trial examining PP in off-pump CABG patients displayed no difference in POAF or pericardial effusion with PP.^24^ A rare complication unique to PP is herniation of posterior coronary grafts through the pericardiotomy, which can lead to arrhythmias, hemodynamic instability, and reoperative intervention; only 1 case of this complication has been reported.^25^

Long-term outcomes after PP are under investigation, and the authors of the PALACS trial are currently investigating long-term mortality or readmission due to cardiovascular causes as a prospective follow-up analysis.^26^ A plausible alternative to PP has been suggested in the form of a posterior pericardial chest tube. In a 2024 study, posterior pericardial chest tube drainage was associated with reduced POAF compared with no posterior pericardial drainage (OR, 0.67; 95% CI, 0.52-0.88; *P* = .003).^27^ However, some surgeons refrain from this technique to avoid mechanical cardiac irritation.^22^ Sen and coworkers^8^ conducted a retrospective assessment of right-sided pericardial window placement (functionally similar to PP) compared with posterior pericardial tube placement. Whereas there were no significant differences in the incidence of POAF, posterior pericardial tube placement was associated with larger pericardial effusions, higher incidence of acute renal failure, and longer intensive care length of stay.^8^

## Comment

The 2005 American College of Chest Physicians set guidelines for the prevention and management of POAF after cardiac surgery, providing a grade B recommendation for the use of PP.^28^ The updated American College of Cardiology and American Heart Association 2023 guidelines on atrial fibrillation management gave a class 2a recommendation for PP for prevention of POAF specifically in patients undergoing CABG, aortic valve, or ascending aortic operations.^29^

Despite these favorable recommendations, PP remains infrequently performed in contemporary practice. Whereas PP is not technically complex, it introduces additional risk, and its long-term outcomes are still uncertain, which may lead surgeons to favor well-established methods such as the use of beta blockers and amiodarone for POAF prophylaxis.^29^ In addition, many studies on the procedure excluded patients with prior cardiothoracic surgeries, dense pleural adhesions, or left ventricular aneurysm, limiting generalizability, which may dissuade surgeons from performing PP, particularly in these populations.^18^

PP is an effective technique for reducing the incidence of cardiac tamponade, pericardial effusions, and POAF in patients undergoing cardiac surgery. Despite an increased occurrence of pleural effusions, PP does not elevate the risk of pulmonary complications, but it can create adhesions to adjacent thoracic structures, which may affect future cardiothoracic surgeries, necessitating individualized surgical planning. Its simplicity and low cost make it a viable option for routine use, pending further integration into clinical practice guidelines. Future research should evaluate the efficacy and safety of alternative techniques including right-sided and bilateral PP, compare these approaches with left PP, and determine applicability across broader populations of patients.
